# Management of diabetes mellitus in patients with chronic kidney disease

**DOI:** 10.1186/s40842-015-0001-9

**Published:** 2015-06-04

**Authors:** Allison J. Hahr, Mark E. Molitch

**Affiliations:** Division of Endocrinology, Metabolism, and Molecular Medicine, Northwestern University Feinberg School of Medicine, 645 N. Michigan Avenue, Suite 530, 60611 Chicago, Illinois USA

**Keywords:** Diabetes, Chronic kidney disease, Diabetic kidney disease, Nephropathy, Glycemic control, Hemoglobin A1c

## Abstract

Glycemic control is essential to delay or prevent the onset of diabetic kidney disease. There are a number of glucose-lowering medications available but only a fraction of them can be used safely in chronic kidney disease and many of them need an adjustment in dosing. The ideal target hemoglobin A1c is approximately 7 % but this target is adjusted based on the needs of the patient. Diabetes control should be optimized for each individual patient, with measures to reduce diabetes-related complications and minimize adverse events. Overall care of diabetes necessitates attention to multiple aspects, including reducing the risk of cardiovascular disease, and often, multidisciplinary care is needed.

## Introduction

Diabetes mellitus is a growing epidemic and is the most common cause of chronic kidney disease (CKD) and kidney failure. Diabetic nephropathy affects approximately 20–40 % of individuals who have diabetes [1], making it one of the most common complications related to diabetes. Screening for diabetic nephropathy along with early intervention is fundamental to delaying its progression in conjunction with providing proper glycemic control. Given the growing population that is now affected by diabetes and thus, nephropathy, knowledge regarding the safe use of various anti-hyperglycemic agents in those with nephropathy is of importance. In addition, attention to modification of cardiovascular disease (CVD) risk factors is essential. Altogether, knowledge regarding the prevention and management of diabetic nephropathy, along with other aspects of diabetes care, is part of the comprehensive care of any patient with diabetes.

## Review

### Recommendations for nephropathy screening in diabetes

Patients with diabetes should be screened on an annual basis for nephropathy. In individuals with type 1 diabetes, screening for nephropathy should start 5 years after diagnosis of diabetes since the onset of diabetes itself is usually known. It typically takes about 5 years for microvascular complications to develop. In patients with type 2 diabetes, screening should begin at initial diagnosis since the exact onset of diabetes is often unknown [1].

Diabetic nephropathy can be detected by the measurement of urine albumin or serum creatinine, and both tests should be performed at minimum annually [1]; those with abnormal levels should have repeat tests done sooner. The first stage of nephropathy is usually the onset of elevated urine albumin which predicts the development of CKD and a gradual decline in glomerular filtration rate (GFR). Some individuals with CKD, however, do not develop elevated urine albumin initially. It is therefore important that individuals have both blood and urine screening tests performed. Using both modalities allows for identification of more cases of nephropathy than using either test alone.

The urine albumin to creatinine ratio can be measured on a spot or timed urine collection such as 4 or 24 h. Microalbuminuria is defined as >30 mg/g creatinine or 30 mg per 24 h. Clinical-or macro-albuminuria is defined as >300 mg/g creatinine or 300 mg per 24 h. An abnormal value should be confirmed on at least one additional urine specimen over a 6 month period. Recently, the terms “moderately increased” and “severely increased” albuminuria have been introduced to replace the terms “microalbuminuria” and “macroalbuminuria”. Increased albumin excretion is not only a marker for early diabetic kidney disease but also for increased risk for macrovascular disease [1]. Other causes of elevated urine protein should be considered and avoided such as infection, strenuous exercise, hypertension, heart failure and hematuria. The serum creatinine should be used to estimate GFR and thus, the level of CKD.

One must also consider that the development of nephropathy may not be related to the diabetes itself. In patients with type 1 diabetes, the onset of retinopathy usually precedes the development of nephropathy. An individual who present with nephropathy but no retinopathy should have an evaluation for other causes. Referral to a nephrologist should be utilized to establish the cause of nephropathy when this is uncertain. Nephrologists are also vital to assist management of complications of advancing kidney disease, such as difficult to control hypertension, hyperkalemia and rapid progression [1, 2].

### Glycemic control in CKD

Glycemic control is essential to delay the onset of complications from diabetes, and it can be challenging for even the most experienced physician. Blood sugar control in those with CKD adds another level of complexity. It requires detailed knowledge of which medications can be safely used and how kidney disease affects metabolism of these medications. In addition, the glycemic target needs to be individualized for each patient, acknowledging that our ability to interpret the data can be altered in the setting of kidney disease.

### Glycemic goal to attain A1c ~7.0 %

Glycemic control is essential to delay or possibly prevent nephropathy. In general, the recommended target A1c for diabetes control by the ADA has been less than or around 7 % [3]. The ADA advises both higher (<8 %) or stricter (<6.5 %) A1c goals for certain populations [3]. AACE suggests a goal A1c of ≤6.5 % in healthy patients who are at low risk for hypoglycemia but also acknowledges the goals need to be individualized [4]. The 2007 Kidney Disease Outcomes Quality Initiative (KDOQI) guidelines for Diabetes and CKD endorse a target A1c of <7.0 % [2] but their updated 2012 guidelines instead recommend an A1c of ~7.0 % [5].

In type 1 diabetes, a number of studies show the development of microalbuminuria is associated with poorer glycemic control. In the DCCT, intensive therapy in patients with type 1 diabetes (mean A1c 9.1 % vs. 7.2 %) reduced the occurrence of microalbuminuria by 34 % in the primary prevention group and 43 % in the secondary intervention group (who had known early complications at baseline); risk reduction in progression to clinical albuminuria was also seen [6, 7]. To assess whether risk reduction of diabetic nephropathy persists long-term, the EDIC Study demonstrated there were fewer cases of new microalbuminuria and progression to albuminuria in the original intensive group. In this long-term follow-up study of the original DCCT treatment groups, it was shown that intensive treatment did result in a significant decrease in the development of estimated GFR levels of <60 ml/min/1.73 m^2^ [8]. In patients with type 2 diabetes, the Kumamoto study, UKPDS and Veterans Affairs Cooperative studies showed reduction of new onset nephropathy and progression of nephropathy with intensive glycemic control [9–11]. A systematic review and meta-analysis of 7 trials evaluating intensive glucose control on kidney-related end points in patients with type 2 diabetes showed lower risk of developing microalbuminuria and macroalbuminuria. The intensive control groups had a median A1c ranging from 6.4–7.4 %. The A1c difference in the intensive groups compared to the control groups ranged from 0.6–2.3 %, with 4 of the studies demonstrating an A1c difference of more than 1 %. The analysis also found there was no benefit in regards to doubling of serum creatinine, development of ESRD or death related to kidney disease [12].

The ACCORD study showed higher risk of hypoglycemia and mortality in patients with type 2 diabetes treated with intensive glucose control (mean A1c 6.4 % vs. 7.5 %), without any risk reduction on CVD. The increased mortality could not be attributed to hypoglycemia [13]. In the ADVANCE trial, more intensive glycemic control (A1c 6.5 % vs. 7.3 %) showed no reduction in CVD. However, the intensive group had a 21 % reduction in nephropathy [14]. The VADT study (intensive group with A1c 6.9 % vs. 8.4 %) also showed no benefit on CVD risk with stricter glucose control [15].

The data clearly show that lowering A1c leads to benefit in regards to nephropathy. Benefits in A1c reduction are also seen on rates of retinopathy and neuropathy. However, the effect of lowering A1c is much less in regards to macrovascular disease. Thus, it is reasonable that a target A1c ~7.0 % offers an optimal risk to benefit ratio rather than a target that is considerably lower.

### Glycemic goal in CKD

Lower A1c levels are associated with higher risk of hypoglycemia which necessitates tailored A1c targets for different individuals. Consequences of hypoglycemia, which in turn can cause injury, myocardial infarction, seizure, stroke or death, are greatest in those who are frail and elderly, with erratic eating habits, on insulin and sulfonylureas, and with CKD. Higher A1c targets should be considered for those with shortened life expectancies, a known history of severe hypoglycemia or hypoglycemia unawareness, CKD, as well as in children.

The Controversies Conference on Diabetic Kidney Disease (DKD) held by KDIGO addressed a number of issues surrounding DKD, including appropriate glycemic control targets [16]. There are insufficient data and trials regarding the ideal glucose target in patients with CKD stage 3 or worse. One study showed that A1c levels >9 % and < 6.5 % were associated with increased mortality in the presence of non-dialysis dependent CKD stage 3 or worse [17]. ESRD patients with diabetes benefit from maintaining their A1c between 7–8 %, as A1c levels above 8 % or below 7 % carry increased risks of all-cause and cardiovascular death [18, 19]. A recent observational study found patients who started dialysis at a younger age (<60 years old) had poorer survival with A1c >8.5 % (HR 1.5 compared to those with A1c 6.5–7.4 %); there was no difference in older patients [20].

### Accuracy of A1c

The hemoglobin A1c can be inaccurate in some patients with kidney disease. Contributing factors include anemia from reduced lifespan of the red blood cell, hemolysis and iron deficiency; falsely increased levels can occur from carbamylation of hemoglobin and the presence of acidosis. Fructosamine and glycated albumin are alternative measures available to estimate glycemic control. Fructosamine reflects the glycation of multiple serum proteins whereas glycated albumin reflects glycation of only albumin; both provide an estimate of control over the past 2 weeks. It is unclear if they offer superior measures of glucose control compared to A1c in patients with CKD. Some studies suggest glycated albumin is superior to A1c in dialysis patients since A1c tends to underestimate glycemic control in those with ESRD, but others argue that A1c remains the gold standard in these patients [21–23].

### Medical therapy in diabetic nephropathy

Medical therapy for diabetes is continually changing as new therapies become available for use and new updates are available that add to our knowledge of the safety profile of available medications. Please refer to Table 1 for adjustments in dosing for diabetes medications used in CKD.

**Table 1:** Dose adjustment for insulin compounds and medications for diabetes in CKD

Medication class	CKD stages 3 and 4 and predialysis stage 5
Insulin	
Glargine	No advised dose adjustment*
Detemir	No advised dose adjustment*
NPH	No advised dose adjustment*
Regular	No advised dose adjustment*
Aspart	No advised dose adjustment*
Lispro	No advised dose adjustment*
Glulisine	No advised dose adjustment*
First-generation sulfonylureas	
Acetohexamide**	Avoid use
Chlorpropamide	eGFR 50–80: reduce dose by 50 %
	eGFR <50: avoid use
Tolazamide	Avoid use
Tolbutamide	Avoid use
Second-generation sulfonylureas	
Glipizide	eGFR <30: use with caution
Glimepiride	eGFR <60: use with caution
	eGFR <30: avoid use
Glyburide	Avoid use
Gliclazide**	No dose adjustment
Glinides	
Repaglinide	No dose adjustment but may wish to use caution with eGFR <30
Nateglinide	eGFR <60: avoid use (but may consider use if patient is on hemodialysis)
Biguanides	
Metformin***	Per FDA, do not use if serum Cr ≥ 1.5 mg/dL in men ≥ 1.4 mg/dL in women.
	Consider
	eGFR ≥45-59: use caution with dose and follow renal function closely (every 3–6 months)
	eGFR ≥30-44: max dose 1000 mg/day or use 50 % dose reduction. Follow renal function every 3 months. Do not start as new therapy.
	eGFR <30: avoid use
Thiazolidinediones	
Pioglitazone	No dose adjustment
Rosiglitazone	No dose adjustment
Alpha-glucosidase inhibitors	
Acarbose	serum Cr >2 mg/dl: avoid use
Miglitol	eGFR <25 or serum Cr >2 mg/dl: avoid use
DPP-4 inhibitor	
Sitagliptin	eGFR ≥50: 100 mg daily
	eGFR 30–49: 50 mg daily
	eGFR < 30: 25 mg daily
Saxagliptin	eGFR > 50: 2.5 or 5 mg daily
	GFR ≤ 50: 2.5 mg daily
Linagliptin	No dose adjustment
Alogliptin	eGFR >60: 25 mg daily
	eGFR 30–59: 12.5 mg daily
	eGFR <30: 6.25 mg daily
SGLT2 inhibitors	
Canagliflozin	eGFR 45 to < 60: max dose 100 mg once daily
	eGFR <45, avoid use
Dapagliflozin	eGFR < 60, avoid use
Empagliflozin	eGFR < 45, avoid use
Dopamine receptor agonist	
bromocriptine mesylate	No dose adjustment known but not studied: use with caution
Bile acid sequestrant	
Colesevelam	No dose adjustment known but limited data
GLP-1 Agonists	
Exenatide	eGFR 30–50: use caution
	eGFR <30: avoid use
Liraglutide	No dose adjustment but use caution when starting or titrating the dose
Albiglutide	No dose adjustment needed
Dulaglutide	No dose adjustment needed
Amylin analog	
Pramlintide	No dose adjustment known but not studied in ESRD

*Adjust dose based on patient response

**Not available in the U.S.

***Recommendations are controversial

### Insulin

Patients with progression of kidney disease are at increased risk of hypoglycemia due to decreased clearance of insulin and some medications used to treat diabetes as well as impairment of renal gluconeogenesis from lower kidney mass. The kidney is responsible for about 30 to 80 % of insulin removal; reduced kidney function is associated with a prolonged insulin half-life and a decrease in insulin requirements as GFR declines [24].

All available insulin preparations can be used in patients with CKD, and there is no specified advised reduction in dosing for patients on insulin. The insulin type, dose and administration must be tailored to each patient to achieve goal glycemic levels but limit hypoglycemia. An inpatient study randomizing weight-based basal and bolus insulin in patients with a GFR <45 mL/min/1.73 m^2^ to 0.5 units/kg body weight vs. 0.25 units/kg showed similar glycemic control but significantly less hypoglycemia in the group with the lower weight-based dose [25].

The **rapid-acting insulin** analogs aspart, lispro and glulisine are the quickest absorbed and are ideal for rapid correction of elevated blood sugars or for prandial insulin needs; they most resemble physiologic insulin secretion. They have an onset of action at 5–15 min, peak action at 30–90 min and an average duration of 5 h. Some studies have shown glulisine has a slightly longer duration of action than the other two rapid-acting insulins. These insulins can be given up to 15 min prior to eating. They are used in “basal-bolus therapy”, also known as multiple daily injections (MDI), as well as in continuous subcutaneous insulin infusions, also known as insulin pumps. The approximate retail cost per vial is $150-165 [26].

Patients with Stage 4–5 CKD and those on dialysis often have some delayed gastric emptying; giving rapid-acting insulin *after* the meal may be helpful for matching the insulin peak with the time of the postprandial blood glucose peak. In patients with nausea who may not know how much they will eat, postprandial rapid-acting insulin dosing may be worth trying. Similarly, patients on peritoneal dialysis obtain large amounts of calories from their dialysis fluid and often eat less than they might expect so that postprandial dosing may be helpful for them also.

The **short-acting insulin** available is regular crystalline insulin, which has an onset of action at 30–60 min, peak action at 2–3 h and duration up to 5–8 h. Regular insulin should ideally be given 30 min prior to a meal. The main advantage of regular insulin is its substantially lower cost compared to the rapid-acting analogs. Regular insulin costs about $90 per vial [26].

The available **intermediate-acting insulin** is isophane, or NPH. It has an onset of action at 2–4 h, peak concentration at 4–10 h and duration up to 10–18 h. In order to achieve adequate basal coverage, it is dosed twice daily. Its use can be limited by its highly variable absorption. Its cost is similar to that of Regular insulin.

The **long-acting insulin** analogs are glargine and detemir. Glargine has an onset of action at 2–4 h, with minimal peak and duration of 20–24 h; it is usually dosed once daily. A unique property of glargine is that it does not have a clear peak. Detemir has an onset of action at 1–3 h, with a small peak at 6–8 h and duration of action of 18–22 h. Detemir is dosed twice daily to give adequate basal coverage in type 1 diabetes; in type 2 diabetes, once daily dosing sometimes is sufficient. The approximate retail price is $160-190 per vial for determir and glargine insulins [26].

There are various **premixed preparation** of insulin that have a fixed percentage of an intermediate-acting and a rapid-or short-acting insulin. Because they contain a combination of 2 insulins, they have two separate peaks. One example is “70/30” which is 70 % NPH and 30 % regular insulin. These preparations offer convenience for the patient with twice daily dosing but offer less flexibility and more restrictions in titration of the insulin. It must be taken at fixed times and the patient must have consistent meals. 70/30 insulin is sometimes helpful in patients getting 12-hours cycled tube feeds.

All insulin is U-100, which is defined as 100 units of insulin/ml. The exception is **insulin U-500** which is 500 units of insulin/ml and is only available as regular insulin. The high concentration of U-500 insulin alters the properties of regular insulin so its pharmacokinetics are different. It has a similar onset of action, near 30 min, but the peak is at 4–8 h and duration is 14–15 h. It can be given up to 30 min prior to meals and is typically given two to three times daily, without the use of a basal insulin [27]. It is generally used in patients who are severely insulin resistant and can be used as a subcutaneous injection or in a pump.

### Oral medications

#### Metformin

Metformin increases insulin sensitivity and decreases hepatic gluconeogenesis; it does not cause hypoglycemia and may lead to weight loss in some patients. It reduces A1c by 1.0–2.0 % [28]. The most common side effects are diarrhea, bloating and cramping. Vitamin B12 deficiency has been reported with extended use [29]. The estimated cost for metformin is about $50 for one month of the 500 mg dose [26].

The FDA recommends that metformin should not be used with serum creatinine ≥ 1.5 mg/dl in men and ≥ 1.4 mg/dl in women or with decreased creatinine clearance in people over age 80. Because metformin is renally cleared, this recommendation is in place to reduce the risk of lactic acidosis in individuals with even modest renal impairment [30]. The overall incidence of lactic acidosis with metformin use, however, appears to be rare. A Cochrane database review of 347 prospective trials and observational cohort studies showed no cases of fatal or non fatal lactic acidosis in 70,490 patient-years of metformin users or in 55,451 patient-years of users of other anti-hyperglycemic agents [31]. In a study evaluating metformin-associated lactic acidosis in 14 patients, other causes of lactic acidosis (including clinical shock or tissue hypoxia) were noted and seemed to be the driving cause and not specifically metformin; 10 of these patients did have metformin accumulation related to elevated serum creatinine (range 3.05-11.8 mg/dl) whereas 4 patients, all with lower creatinine levels though still reduced GFR, had no evidence of metformin accumulation [32].

Given the differences in translation of creatinine into creatinine clearance based on age, weight and race, it is reasonable to consider use of a GFR-based guideline such as outlined here rather than one based on creatinine alone. Metformin can be used without dose reduction with an eGFR >60 ml/min/1.73 m^2^. If the eGFR is ≥45–59 ml/min/1.73 m^2^, it is prudent to continue use of metformin but take caution with dosing and follow the renal function more closely, such as every 3 to 6 months. If the eGFR is ≥30–44 ml/min/1.73 m^2^, again use caution with dosing, such as limiting its dose to a maximum of 1000 mg daily or using a 50 % reduction, follow renal function every 3 months and avoid newly initiating metformin in patients with this level of CKD. Metformin should be avoided with eGFR <30 ml/min/1.73 m^2^. It is recommended that metformin be stopped in the presence of situations that are associated with hypoxia or an acute decline in kidney function such as sepsis/shock, hypotension, acute myocardial infarction, and use of radiographic contrast or other nephrotoxic agents [33, 34]. This approach has been accepted by various societies including KDIGO and confirmed in additional studies [35] [36]. The KDIGO Controversies Conference proposed a change to the FDA guidelines [16].

#### Sulfonylureas

Sulfonylureas bind to the sulfonylurea receptor on the pancreatic beta-cells and lead to increased insulin secretion. They typically lower A1c by 1.5–2 % and can cause hypoglycemia. The first-generation sulfonylureas are rarely prescribed. The second-generation sulfonylureas, which include glipizide, glimepiride, glyburide, and gliclazide (the latter is not available in the U.S.), are commonly used. The sulfonylureas will decrease A1c by 1–2 % [28]. The estimated cost for one month of glipizide and glyburide (5 mg) and glimepiride (2 mg) ranges from $10 to $30 [26].

Sulfonylureas and their metabolites are renally cleared, leading to an increased risk of hypoglycemia as GFR declines. Hypoglycemia is greatly increased with glimepiride and glyburide with GFR <60 ml/min/1.73 m^2^ due to the presence of two active metabolites cleared in part by the kidney [37]. Glyburide should be avoided with eGFR <60 ml/min/1.73 m^2^ [38]. Glimepiride should be used with caution if the eGFR is <60 ml/min/1.73 m^2^ and not be used with eGFR <30 ml/min/1.73 m^2^ [37]. Less than 10 % of glipizide is cleared renally but it should still be used with caution with an eGFR <30 ml/min/1.73 m^2^ due to the risk of hypoglycemia [39, 40].

#### Glinides

Nateglinide and repaglinide, like sulfonylureas, increase insulin secretion by closing a sulfonylurea receptor/ATP-dependent potassium channel on the beta-cells of the pancreas. They have a shorter half-life compared to the sulfonylureas. They result in a rapid and short duration of insulin release and should be taken prior to meals. They also can cause hypoglycemia [41]. The glinides reduce A1c on average by 0.5–1.5 % [28] and have an estimated cost of $90 per month (for repaglinide 1 mg, and $60 per month for nateglinide 120 mg) [26].

The active metabolite of nateglinide accumulates in CKD; nateglinide should not be used with an eGFR <60 ml/min/1.73 m^2^. The active metabolite is cleared, however, by hemodialysis so nateglinide can be used in those undergoing dialysis [42]. Conversely, repaglinide appears safe to use in individuals with CKD [43]. However, it is reasonable to exercise caution in those with more severe renal dysfunction, such as an eGFR <30 ml/min/1.73 m^2^, and start at the lowest dose (0.5 mg) with slow upwards titration.

#### Thiazolidinediones

Thiazolidinediones (pioglitazone, rosiglitazone) increase insulin sensitivity by acting as PPARγ agonists. They do not cause hypoglycaemia and they lead to an A1c decrease of 0.5–1.4 % [28]. They are metabolized by the liver and can be used in CKD. However, fluid retention is a major limiting side effect and they should not be used in advanced heart failure. This also makes their use in CKD, particularly patients on dialysis, limiting. They have been linked with increased fracture rates and bone loss, thus use in patients with underlying bone disease (such as renal osteodystrophy) needs to be considered. No dose adjustment is indicated with either in CKD. One month of 15 mg of pioglitazone costs about $260 and 2 mg of rosiglitazone costs about $100 [26]. In September 2010, the FDA restricted use of rosiglitazone based on studies linking it to increased cardiovascular events. Upon further review, these restrictions were lifted in 2014.

An association between pioglitazone and bladder cancer has been raised but further analysis and investigation into the data shows that this association is not clearly supported [44]. A recent pooled multi-population analysis also showed no association between the thiazolidinediones and bladder cancer [45].

#### Alpha-glucosidase inhibitors

Alpha-glucosidase inhibitors (acarbose, miglitol) decrease the breakdown of oligo-and disaccharides in the small intestine, slowing ingestion of carbohydrates and delaying absorption of glucose after a meal. The major side effects are bloating, flatulence, and abdominal cramping. They typically lower A1c by 0.5–0.8 % and usually do not lead to weight gain or loss [28]. The approximate cost for one month of 25 mg of either dose is about $30 (acarbose) to $250 (miglitol) [26].

Acarbose is minimally absorbed with <2 % of the drug and active metabolites present in the urine. With reduced renal function, serum levels of acarbose and metabolites are significantly higher. Miglitol has greater systemic absorption with >95 % renal excretion. It is recommended that use of miglitol be avoided if the GFR is <25 ml/min/1.73 m^2^ [46]. Additionally, neither medication has been studied long-term in patients with a creatinine >2 mg/dl, so their use should be avoided in these patients.

#### Dipeptidyl peptidase-4 inhibitors

Dipeptidyl peptidase 4 (DPP 4) inhibitors decrease the breakdown of incretin hormones such as GLP-1 and include sitagliptin, saxagliptin, linagliptin, and alogliptin. This class of medication is weight-neutral and decreases A1c by 0.5–0.8 % [28]. One month of 50 mg sitaglipitin or 5 mg saxagliptin is about $280 [26].

Approximately 80 % of sitagliptin is cleared by the kidney; with an eGFR of ≥30 to <50 ml/min/1.73 m^2^, 50 mg once daily should be used and with an eGFR <30 ml/min/1.73 m^2^, a dose of 25 mg once daily isadvised [47]. Saxagliptin also needs a dose reduction with eGFR ≤ 50 ml/min/1.73 m^2^ to 2.5 mg daily; otherwise, the standard dose with eGFR >50 ml/min/1.73 m^2^ is 2.5 or 5 mg daily. Only a small amount of linagliptin is cleared renally; thus, no dose adjustment is indicated with a reduced GFR [48]. Alogliptin also needs a dose reduction from the baseline dose of 25 mg daily to 12.5 mg daily with an eGFR < 60 ml/min/1.73 m^2^ and then to 6.25 mg daily with an eGFR < 30 ml/min/1.73 m^2^.

#### Sodium-glucose co-transporter 2 (SGLT2) inhibitors

SGLT2 inhibitors reduce glucose absorption from the kidney, leading to an increase in glucose excretion and a reduction in A1c of about 0.9–1.0 % [49]. The increase in urine glucose can result in a weight loss of up to 5 kg in one year. Because of an increase in adverse events related to intravascular volume contraction, no more than 100 mg once daily of canagliflozin should be used in patients with an eGFR of 45 to < 60 ml/min/1.73 m^2^. Its use should be avoided if the eGFR is <45 ml/min/1.73 m^2^ because of an increase in adverse events as well as reduced efficacy. Dapagliflozin is not approved for use if the eGFR is < 60 ml/min/1.73 m^2^ but empagliflozin can be used down to an eGFR of 45 ml/min/1.73 m^2^ ml/min/1.73 m^2^. Costs for 30 days of the lowest doses of these drugs are in the $350–400 range.

#### Other oral medications

Bromocriptine (dopamine receptor agonist) has not been adequately studied in CKD.

Colesevelam (bile acid sequestrant) shows no difference in efficacy or safety in those with an eGFR <50 ml/min/1.73 m^2^ but data are limited as it has not been adequately studied in more advanced CKD. A one month supply of the 625 mg tablets (6 tablets per day must be taken) is about $420.

### Other subcutaneous medications

#### Glucagon-like peptide 1 (GLP-1) Receptor Agonists

Exenatide (regular and extended-release) and liraglutide are injectable medications that mimic gut hormones known as incretins, leading to insulin release, delayed glucagon secretion and delayed gastric emptying. They are FDA approved for use with metformin and/or sulfonylureas although in practice, they are also used with insulin. They contribute to central satiety leading to a reduction in appetite and often weight loss. The average expected A1c decrease is 0.5–1.0 % [28]. The costs of exenatide regular-release is about $385 for a 10 mcg pen and $596 for 3 pens of the liraglutide [26]. Both agents have been associated with pancreatitis, and nausea is a common side effect that can limit its use. In addition, liraglutide has been associated with the development of thyroid C-cell tumors in animal studies and thus should not be given to patients with or at risk for medullary thyroid cancer. Exenatide is given twice daily and liraglutide is given once daily; exenatide extended-release is dosed once weekly. Albiglutide and dulaglutide are other GLP-1 receptor agonists that can also be dosed once weekly.

Clearance of exenatide decreases with declines in GFR [50]. Additionally, in a case report of a patient with renal impairment and CKD, use of exenatide led to a rise in serum creatinine that resolved when the medication was stopped [51]. The FDA reported cases of acute renal failure associated with exenatide use and recommends it be used with caution in those with a GFR of 30–50 ml/min/1.73 m^2^ and not be used if the GFR is <30 ml/min/1.73 m^2^ [52]. Liraglutide is not metabolized primarily by the kidney; no dose adjustment is indicated in those with renal impairment, including ESRD, although data in this population are limited [53]. No dosage restrictions are needed for albiglutide or dulaglutide with decreasing GFR [54, 55]. The manufacturer has reported cases of renal failure and worsening of chronic renal impairment with its use and advises caution with initiating or increasing the dose in those with nephropathy.

#### Amylin analog

Pramlintide is also an injectable medication that is used with meals as an adjunct to insulin therapy in both type 1 and type 2 diabetes. Amylin is secreted along with insulin by pancreatic beta-cells and levels are low in patients with diabetes. It typically reduces A1c by 0.5–1.0 % [28] with a cost of about $400 for two of the 1.5 mL pens (1000 mcg/mL) [26]. No dose adjustment appears necessary for CKD; it has not been studied in ESRD.

### Strategy for glycemic control and other risk factors

The primary goal of optimizing glycemic control to reduce the development of microvascular and macrovascular complications is universal. The medication regimen is based on the comfort of the patient and physician and should be individualized, especially as renal function changes.

For those who need insulin, MDI with an average of 4 daily injections is common. The closest approximation of physiologic insulin secretion can be achieved with an insulin pump delivering a continuous subcutaneous infusion. A single type of insulin is used in the pump such as a rapid-acting analog that serves as the basal, bolus and correction insulin. Insulin pumps require vigilance on the part of the patient and their use should be overseen by endocrinologists and experienced diabetes educators.

Continuous Glucose Monitoring Systems (CGMS) are available that can continually measure glucose levels. A small plastic catheter is inserted subcutaneously and measures glucose every 5 min. Patients can view this in real-time and detect upward and downward trends in glucose. The added benefit is that alarms for high and low readings can be set.

In addition to glucose control, a comprehensive approach to care is encouraged. Behavioral modification and lifestyle changes are important to control weight, improve nutrition, modify dietary intake and monitor glucose levels. Appropriate medication should be used for treatment of nephropathy, in conjunction with a nephrologist as appropriate. Close attention should also be paid to blood pressure control. Diabetes in itself is a major cause of cardiovascular disease and individuals with CKD often die of CVD; it is the major cause of death in this population. The presence of microalbuminuria, albuminuria and declining GFR are all known predictors of CVD. The combination of diabetes and CKD is particularly powerful in regards to CVD risk, necessitating aggressive control of risk factors [56]. In addition to hypertension, dyslipidemia and weight control should be addressed. Nutrition plays an important role in individuals with diabetic kidney disease as a balance of multiple dietary factors including sodium, potassium, phosphorus, and protein intake must be followed as well as intake of carbohydrates and unhealthy fats. Reduction in weight in patients who are overweight or obese and increases in exercise are generally recommended, keeping in mind the need for cardiac stress testing. It is helpful to use an experienced dietician and certified diabetes educator to safely attain dietary, exercise and weight loss goals. The KDIGO Controversies Conference addresses some of the issues surrounding diabetic kidney disease management including management of dyslipidemia and blood pressure control [16]. The American Diabetes Association also has recommendations on management of blood pressure and dyslipidemia [57].

### Medical therapy in dialysis and post-transplant patients

There are a few oral agents that can be used safely in patients on dialysis, particularly if the diabetes is fairly mild. Most others, however, will need insulin for glycemic control.

Patients receiving hemodialysis (HD) can have different clearance rates of insulin that may be affected by the timing of dialysis. We have done continuous glucose monitoring on patients undergoing HD and found that patients’ glycemic responses during HD are quite idiosyncratic and their insulin regimens need to be individualized to avoid both hyper-and hypoglycemia during and after HD. Patients who are on peritoneal dialysis (PD) have exposure to large amounts of glucose in the dialysate that can lead to uncontrolled hyperglycemia. In patients receiving PD continuously, a standard basal/bolus insulin regimen is best. However, with overnight PD using a cycler, coverage of the increased glucose load may best be accomplished using a fixed mixture insulin combination, such as 70/30 or 75/25 insulins, given at the onset of PD. The nephrologist prescribing the PD will often change the glucose concentration of the dialysate because of the need for more or less fluid removal and such changes need to be discussed with the endocrinologist so that the insulin doses may be appropriately changed.

In the immediate post-transplant period, glycemic control can acutely decline. This is due to the initiation of anti-rejection therapies including glucocorticoids, calcineurin inhibitors and sirolimus, and an increase in insulin resistance. In addition, patients may experience other fluctuations in their daily routines including adjustments in diet, activity and medications. Because many variables are present, glycemic control can fluctuate quite a bit, and close monitoring of blood glucose levels and adjustments of medications are needed.

## Conclusions

The management of patients with diabetes and nephropathy necessitates attention to several aspects of care. Importantly, glycemic control should be optimized for the patient, attaining the necessary control to reduce complications but done in a safe, monitored manner. Screening for development of nephropathy should be performed on a regular basis to identify microalbuminuria or reductions in GFR and if identified, the diabetes regimen should be tailored accordingly. Prevention and treatment of diabetic nephropathy and other complications necessitates a multifactorial approach through the use of a diabetologist, nephrologist, dietician, diabetes educator and additional specialists experienced in the complications of diabetes to provide a multifaceted care program to reduce progression of disease.

## Abbreviations

CKD: Chronic kidney disease
CVD: Cardiovascular disease
GFR: Glomerular filtration rate
DKD: Diabetic kidney disease
MDI: Multiple daily injections
CSII: Continuous subcutaneous insulin infusion
DPP4: Dipeptidyl peptidase-4 inhibitors
SGLT2: Sodium-glucose co-transporter 2
GLP1: Glucagon-like peptide 1
HD: Hemodialysis
PD: Peritoneal dialysis
